# Preclinical pharmacological profile of nomegestrol acetate, a synthetic 19-nor-progesterone derivative

**DOI:** 10.1186/1477-7827-10-85

**Published:** 2012-10-08

**Authors:** Harry A van Diepen

**Affiliations:** 1 Women’s Health Department, Merck Sharp & Dohme (MSD) Corp., Oss, The Netherlands

**Keywords:** Nomegestrol acetate (NOMAC), Progesterone, Oral contraceptives, Preclinical studies, Pharmacology

## Abstract

**Background:**

Nomegestrol acetate (NOMAC), a synthetic progestogen derived from 19-nor-progesterone, recently completed clinical trials for use with 17beta-estradiol in a new monophasic combined oral contraceptive. In this review, published as well as previously unpublished preclinical studies that detail the effects of NOMAC on estrogenic, progestogenic, and androgenic systems, as well as mineralocorticoid, glucocorticoid, bone, and metabolic indices are described.

**Methods:**

*In vitro* assays to determine NOMAC structure-activity relationships used tissue derived from rat uteri. Transactivation profiles were performed using Chinese hamster ovary (CHO) cells transfected with cDNAs encoding human steroid receptors. Estrogenic and anti-estrogenic activities were monitored *in vivo* in rats as well as *in vitro* in human breast cancer cells. Standard *in vivo* techniques were used in rats to determine progestational activity; antigonadotropic, androgenic, mineralocorticoid, and glucocorticoid activities; as well as effects on bone and other metabolic indices. Ovulation inhibition was monitored in rats and primates. NOMAC’s effects on cardiovascular systems were determined in dogs and primates.

**Results:**

NOMAC was without significant agonistic or antagonistic activity for estrogen receptor alpha or beta *in vitro*, and inhibited ovulation in rats and monkeys (2.5 mg/kg and 1 mg/kg, respectively). NOMAC lacked androgenic, antimineralocorticoid, glucocorticoid, and metabolic activity and exhibited moderate anti-androgenic activity in rats. NOMAC did not affect bone mineral density (BMD) in rats or hemodynamic and electrophysiologic parameters in dogs and primates.

**Conclusions:**

NOMAC is a selective progestogen structurally similar to progesterone that has modest anti-androgenic activity and does not affect lipid or carbohydrate metabolism, BMD, or many cardiovascular parameters in selected animal models.

## Background

**Figure 1:**
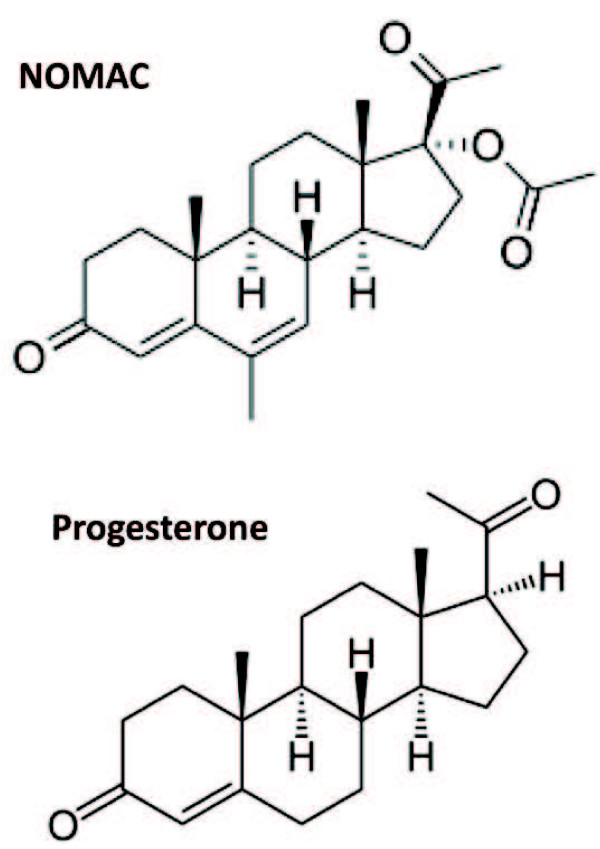
**Structures of progesterone [**[2]**] and NOMAC [**[3]**].**

Most combined oral contraceptives (COCs) in use today contain a synthetic progestogen (predominantly 19-nortestosterone derivatives) and a synthetic estrogen, ethinyl estradiol (EE). In the early 1980s, nomegestrol acetate (NOMAC), a progestogen derived from 19-nor-progesterone, was synthesized (Figure 1) [1-3]. Although used clinically for treatment of climacteric symptoms [4], NOMAC was recently combined with 17β-estradiol (E2) to be used in a new COC [5-10].

## Methods

In this article, we present both previously published and unpublished data to deliver a complete preclinical pharmacological profile of NOMAC. The studies were approved by the review board for animal experimentation at Merck Sharp & Dohme (MSD, Oss, The Netherlands) or at Theramex (Monaco) and were performed in accordance with the principles and procedures described in the European Union guidelines for the care and use of laboratory animals. Data are reported as ± standard error of the mean unless otherwise indicated. Comparisons between groups were analyzed using analysis of variance (ANOVA) models, unless otherwise indicated and statistical significance was defined as *P*<0.05.

## Results

### *In vitro* progestogenic activity

The *in vitro* progestogenic activity of NOMAC was initially assessed by studying its structure-activity relationship with specific ligands on several cytosolic steroid receptors [11]. The relative binding affinity (RBA) of NOMAC for the rat uterine progesterone receptor (PR) is very similar to the binding affinity of progesterone. After 2 and 24 hours of incubation with rat uterine PR, the RBA of NOMAC (relative to progesterone) was 72% and 92%, respectively.

Duc *et al.*[12] determined the apparent binding constant at equilibrium (*k*_*d*_), maximum concentration of binding sites (*B*_*max*_), the dissociation rate constant *k*_*−1*_, and the specific RBA (relative to progesterone) of radiolabeled NOMAC to PR derived from the uteri of estrogen-primed rats. In their studies, NOMAC had high specificity for rat uterine PR; saturation occurred at approximately 30 nM. Nonspecific binding was very low, accounting for only 5-7% of total binding. Determination of the specific binding constants indicated that NOMAC had a *k*_*d*_ and *B*_*max*_ of 5.44 ± 1.27 nmol/L and 1.51 ± 0.11 pmol/mg protein, respectively. In addition, it was determined that NOMAC binds avidly, but is slow to dissociate from rat uterine PR (*k*_*−1*_ = 4.9 ± 0.5 × 10^–5^ s^–1^).

### Transactivation profile

Receptor-binding experiments, although useful, do not distinguish between agonistic and antagonistic activity. van Diepen *et al.*[13] used established cellular assays [14-16] to determine the agonistic and antagonistic profiles of NOMAC, levonorgestrel (LNG), drospirenone (DRSP), dienogest (DNG), and progesterone in Chinese hamster ovary (CHO)-K1 cells that were stably transfected with cDNAs encoding human receptors, including progesterone receptor B (hPRB), androgen receptor (hAR), mineralocorticoid receptor (hMR), glucocorticoid receptor (hGR), and estrogen receptors (ER) α and β (hER_alpha_ and hER_beta_, respectively). NOMAC and LNG were the most potent progestogens in activating hPRB in CHO cells. NOMAC did not display antagonistic activity against hPRB. NOMAC, DRSP, DNG, LNG, and progesterone were all capable of antagonizing hAR. None of the progestogens tested (including NOMAC) activated or antagonized hER_alpha_, hER_beta,_ or hGR or were hMR agonists. In addition, NOMAC did not have antimineralocorticoid activity. These experiments support the overall conclusion that NOMAC has a progestational profile that is similar to progesterone.

### *In vivo* progestational activity

Progestogens antagonize estrogen action by induction of estradiol metabolism and down-regulation of the ER [17-20]. Progesterone down-regulation of the ER and the agonistic versus antagonistic effect of progesterone on uterine weight is dependent on the concentration of plasma E2 [21]. Moreover, progesterone is far more effective in reducing ER levels in estrogen-primed tissue [22].

The effects of NOMAC treatment on the expression of cytosolic uterine ER were studied in Sprague–Dawley ovariectomized (OVX) rats. After recovering from ovariectomy for at least 15 days, each rat received an intraperitoneal injection of vehicle or E2 (5 μg/rat). After 24 hours, each rat received either vehicle (n = 8), E2 only (n = 6), E2 + progesterone (100, 250, or 950 μg/rat; n = 5 per dose), E2 + NOMAC (100, 250, or 950 μg/rat; n = 5 per dose), or E2 + medroxyprogesterone acetate (MPA; 100, 250, or 950 μg/rat; n = 5 per dose). After an additional 24 hours, the rats were killed and uteri removed. ER contained in nuclear cell fractions originating from rat uterine cytosol were determined using a ^3^[H] E2 exchange assay [21,23].

**Figure 2:**
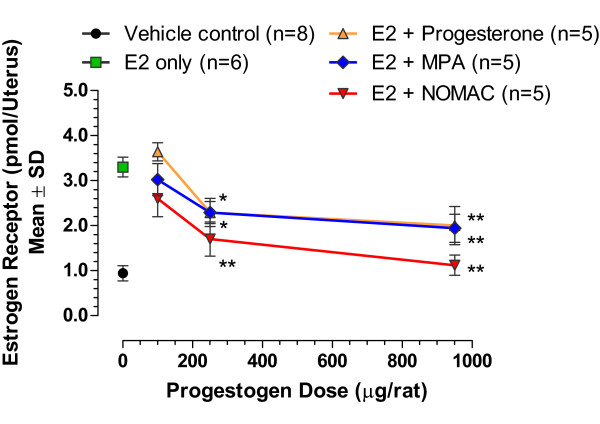
**Inhibition by progestogens of estrogen-stimulated uterine estrogen receptor content in OVX rats.** ANOVA, analysis of variance; OVX, ovariectomized; E2, 17β-estradiol; NOMAC, nomegestrol acetate; MPA, medroxyprogesterone acetate; SD, standard deviation. * *P*<0.05; ** *P*<0.01 compared with E2-only treated rats (ANOVA).

The administration of E2 alone increased the quantity of ER in the uterus by 3-fold (Figure 2). Progesterone, NOMAC, and MPA caused a dose-dependent inhibition of E2-stimulated ER. When administered alone (without E2), NOMAC, MPA, and progesterone did not influence uterine ER. This study demonstrated that NOMAC acted *in vivo* to inhibit ER expression in E2-primed rat uterus.

### Antigonadotropic activity and ovulation inhibition

Paris *et al.*[1] and van Diepen [13] investigated the ability of NOMAC to inhibit ovulation in the rat. After 3 consecutive, regular 4-day cycles, animals in the metestrus phase received single subcutaneous injections of NOMAC or progesterone at doses of 0.25, 1, or 4 mg (n = 5-6 per dosage group) or vehicle (n = 17). In this study, the rate of ovulation in the vehicle-treated control animals was 76.5%. At the lowest dose (0.25 mg/rat), NOMAC inhibited ovulation in 78.2% (5/6) of rats, indicating that the 50% inhibition dose (ID50) for NOMAC was <1.25 mg/kg. The ID50 for progesterone was approximately 5 mg/kg. Oral NOMAC produced similar results and suggested that oral NOMAC is at least 2 times more potent than oral LNG in inhibiting ovulation in rats. In *Cynomolgus* monkeys (*Macaca fascicularis*), which have a menstrual cycle very similar to the human menstrual cycle (average *Cynomolgus* cycle length, 29 days; range, 26-32 days), NOMAC was also able to inhibit ovulation. NOMAC administered for 1 cycle to 34 *Cynomolgus* monkeys provided effective, dose-dependent inhibition of ovulation, which was observed in 4/11, 7/10, and 11/11 monkeys (0.10, 0.25, and 1.00 mg/kg/day NOMAC, respectively) [13]. The ID50 of NOMAC was 0.14 mg/kg. Treatment with NOMAC did not influence body weight or cycle length relative to nontreatment.

### *In vitro* estrogenic and anti-estrogenic activity

Estrogens stimulate proliferation of human breast tumor cells [24]. However, the effect of progestogens on human breast tumor cells is more controversial. Progestogens that interact with the ER can have a stimulatory effect growth of T-47D and MCF-7 cells [25], whereas progestogens that do not interact with ER do not stimulate cell proliferation. To determine the effect of NOMAC on breast cancer cell growth, in the absence of E2, Catherino *et al.*[26] investigated the effect of NOMAC and megestrol acetate (MAC) on growth of MCF-7 and T-47D cells. The cells were incubated for 6 days in the presence of NOMAC or MAC at doses up to 10^–6^ M and the proliferative activity of cells grown in the presence of progestogen was determined (measured by total DNA). As would be expected of progestogens that do not activate ER, NOMAC and MAC at doses up to 1 μM/L did not increase proliferative activity.

Although NOMAC has no growth-stimulating activity on breast cancer cells in the absence of E2, some evidence suggests that it may also help attenuate the proliferative effects of endogenous E2 by preventing E2 formation from estrone sulfate (E1S) and by enhancing the conversion of E2 to less potent metabolites. In support of this possibility, NOMAC was able to inhibit estrone sulfatase and 17β-hydroxysteroid dehydrogenase activity, 2 enzymes responsible for the conversion of E1S to E2 [27]. In T-47D cells, NOMAC (5 × 10^–6^ M and 5 × 10^–5^ M) blocked the conversion of E1S (2 × 10^–6^ M) to E2 by –60% and –71%, respectively. In MCF-7 cells, NOMAC (5 × 10^–6^ M and 5 × 10^–5^ M) blocked the conversion of E1S (5 × 10^–9^ M) to E2 by –43% and –77%, respectively.

NOMAC also had a stimulatory effect on the activity of estrogen sulfotransferase, an enzyme that converts E1 to E1S or E2 to E2S in hormone-dependent MCF-7 and T-47D human breast cancer cells [28]. At concentrations of 5 × 10^–8^ M and 5 × 10^–7^ M, NOMAC increased the activity of sulfotransferase in MCF-7 cells by 60.6% and 83%, respectively. In T-47D cells, NOMAC (5 × 10^–7^ M) increased estrogen sulfotransferase activity by 69.2%. Taken together, these studies indicate that NOMAC alone does not induce breast cancer cell growth *in vitro* and may reduce the proliferative effect of E2 in these cells by preventing conversion of E1S to more potent E2.

### *In vivo* estrogenic and anti-estrogenic activity

**Figure 3:**
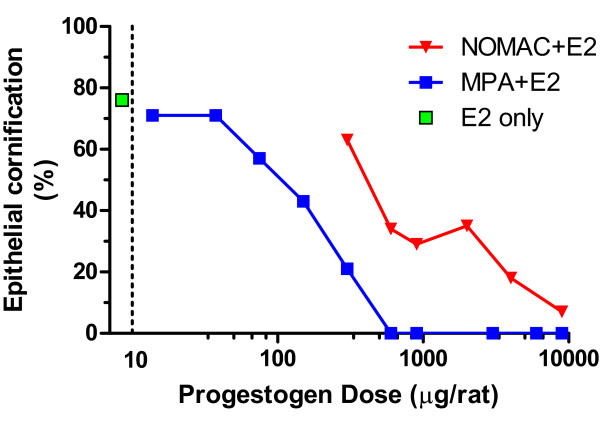
**Effect of NOMAC and MPA on E2-stimulated vaginal epithelial cornification in OVX rats.** OVX, ovariectomized; E2, 17β-estradiol; NOMAC, nomegestrol acetate; MPA, medroxyprogesterone acetate.

Although NOMAC does not bind to or transactivate ER, several experiments were performed to evaluate possible estrogenic activity *in vivo* that could be mediated by actions at the PR. To test this possibility, Sprague–Dawley OVX rats (90-100 g) were treated with a single subcutaneous injection of E2 (5 μg/rat) followed by oral vehicle (n = 47; control; no progestogen), oral NOMAC (n = 178; 0.3-9.0 mg/rat), or oral MPA (n = 84; 0.014-9.0 mg/rat). The percentage of rats in each group with positive vaginal smears (indicating epithelial cornification) at 56 and 72 hours after treatment with E2 was determined. Epithelial cornification occurred in 76.6% of control rats (36/47 in estrus) that received E2 only (no progestogen). NOMAC and MPA produced dose-dependent inhibition of epithelial cornification (Figure 3), a strong indicator of anti-estrogenic activity. The ID50 values for NOMAC and MPA were 450 and 92 μg/rat, respectively. A small dose of NOMAC (0.6 mg/rat) was enough to inhibit epithelial cornification in approximately two-thirds of rats. These experiments demonstrated that NOMAC had only anti-estrogenic activity and prevented E2-induced vaginal cornification in OVX rats in a dose-dependent manner.

The anti-estrogenic activity of NOMAC was further investigated in mice using a method devised by Edgren [29]. Three-week-old mice (8-11g) received subcutaneous injections of estradiol benzoate (0.1 μg/3 days) and subcutaneous injections of progesterone, MPA, or NOMAC (0.3, 0.6, and 0.9 mg/mouse/3 days for each compound). In a separate study, mice received subcutaneous injections of estradiol benzoate (0.1 μg/3 days) and oral doses of MPA (0.3, 0.6, or 0.9 mg/mouse/3 days) or NOMAC for 3 consecutive days (0.1, 0.3, 0.6, 0.9, or 1.6 mg/mouse/3 days). At 24 hours after the last dose, the uteri were removed, weighed, and dissected. In estradiol-benzoate-treated mice, subcutaneous NOMAC (0.6 and 0.9 mg) significantly decreased the weight of the uterus compared with control mice that received estradiol-benzoate but no progestogen (Table 1). High doses of subcutaneous progesterone (0.9 mg) did not decrease uterine weight significantly. Although a moderate subcutaneous dose of MPA (0.6 mg) was able to induce a statistically significant decrease in uterine weight, the uteri of mice receiving the lowest and highest dosages of MPA did not undergo statistically significant weight reductions. Oral MPA (0.3-0.9 mg) did cause statistically significant reductions to the weight of the uterus (Table 1). In addition, oral NOMAC (0.3-1.8 mg) administered over a 3-day period induced a significant reduction in the weight of the uterus compared with control mice (subcutaneous estradiol-benzoate treatment only; no progestogen). These experiments demonstrated that NOMAC administration had a significant anti-estrogenic effect in estradiol-benzoate-treated mice.

**Table 1:** Effect of progesterone, MPA, and NOMAC on the uterine weight in estradiol-benzoate-treated mice

**Estradiol benzoate**	**Progestogen**	**Dose**	**No. of mice**	**Uterine weight**
		**(mg/mouse/3 days)**		
*Subcutaneous administration*^*a*^				
–	–	–	20	21.61 ± 1.37**
0.1	–	–	10	32.88 ± 2.26
0.1	Progesterone	0.3	5	32.04 ± 1.38
0.1		0.6	5	30.46 ± 1.53
0.1		0.9	5	29.78 ± 2.99
0.1	MPA	0.3	5	27.50 ± 1.08
0.1		0.6	5	24.92 ± 2.37*
0.1		0.9	5	29.34 ± 2.42
0.1	NOMAC	0.3	5	27.16 ± 3.22
0.1		0.6	5	23.72 ± 1.08**
0.1		0.9	5	23.94 ± 1.16**
*Oral administration*^*a*^				
–	–	–	40	12.31 ± 0.49
0.1	–	–	31	40.99 ± 2.11
0.1	MPA	0.3	8	30.08 ± 3.92*
0.1		0.6	7	22.39 ± 3.09**
0.1		0.9	8	24.70 ± 2.27**
0.1	NOMAC	0.1	9	46.74 ± 4.48
0.1		0.3	16	29.22 ± 1.89**
0.1		0.6	28	30.97 ± 2.47**
0.1		0.9	16	25.07 ± 2.28**
0.1		1.8	9	25.09 ± 4.37**

Uterine weights (mg) are expressed as the mean ± standard deviation.

^a^Estradiol-benzoate was administered as a subcutaneous injection. *MPA*, medroxyprogesterone acetate. * *P*<0.01; ** *P*<0.001 compared with the estradiol-benzoate-treated control group.

### Androgenic and anti-androgenic activity

Several studies have demonstrated that NOMAC is devoid of androgenic activity [1,13,30,31]. In some of the initial experiments to examine androgenic activity, sexually immature, castrated male rats were treated with a high dose of NOMAC (10 mg/rat) for 10 days; it did not increase the weights of male accessory sex organs [31] or have other androgenic or anabolic effects [1]. Although these studies showed that NOMAC did not possess androgenic activity in rats, it did have significant anti-androgenic activity, which was estimated to be between 3 to 5 times greater than that of chlormadinone acetate (CMA) [1]. In transactivation studies, NOMAC was also found to be an effective antagonist against hAR [13], with much greater anti-androgenic activity than progesterone, DNG, and DRSP, but less activity than LNG. These results were consistent with previous findings demonstrating that the binding affinity of NOMAC for rat androgen receptor was approximately 4 times greater than that of progesterone [30].

**Figure 4:**
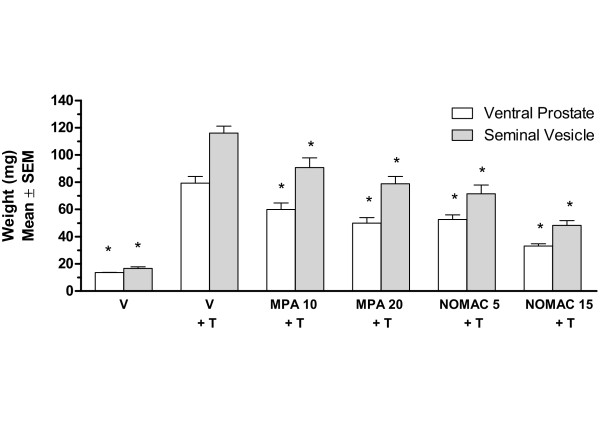
**Antiandrogenic activity of NOMAC and MPA in castrated mature rats.** Rats were treated with vehicle (V), V + testosterone propionate (T; 100 μg/day), T + NOMAC (5 or 15 mg/day), or T + MPA (10 or 20 mg/day) for 7 days and the weights of the ventral prostate and seminal vesicles determined. ANOVA, analysis of variance; NOMAC, nomegestrol acetate; MPA, medroxyprogesterone acetate. * *P*<0.001 vs V + T treated controls (ANOVA).

A previously unpublished study in mature orchidectomized rats demonstrated the anti-androgenic effects of NOMAC on male accessory organs *in vivo* (Figure 4). In this study, oral NOMAC (5 or 15 mg/day) or MPA (10 or 20 mg/day) or vehicle was co-administered with testosterone propionate (100 μg/day) for 7 days. The weights of the ventral prostate and seminal vesicles decreased significantly in animals that received NOMAC or MPA relative to testosterone propionate-treated controls, results that were consistent with previously published data comparing NOMAC and cyproterone acetate [31]. In sexually mature, intact male rats that did not receive exogenous testosterone, oral NOMAC (5 or 15 mg/rat/day), or MPA (10 or 20 mg/rat/day) for 12 days also resulted in significant weight reductions of the ventral prostate and seminal vesicles, additional evidence of anti-androgenic activity (data not shown).

### Glucocorticoid and antiglucocorticoid activity

Botella *et al.*[11] assessed the *in vitro* glucocorticoid activity of NOMAC and other synthetic steroids relative to a known glucocorticoid receptor (GR) agonist (dexamethasone) and found that NOMAC had very little interaction with rat liver or kidney GR. NOMAC incubated with rat liver GR for 2 hours had a half maximal inhibitory concentration (IC50) of 167 ± 21 nmol/L (10.3%) relative to dexamethasone (IC50, 17.2 ± 3.8 nmol/L). Although the IC50 of dexamethasone was unchanged after 24 hours, the IC50 of NOMAC increased to 1493 ± 807 nmol/L (1%). These results demonstrate that NOMAC has less affinity for rat liver GR over time. These results suggest a low association and faster dissociation to rat GR of NOMAC compared with dexamethasone. However, this effect was not observed when testing the glucocorticoid effect of NOMAC on hGR.

The effect of NOMAC and comparator progestogens on the pituitary-adrenal axis was assessed in OVX rats by measuring plasma corticosterone and the weight of the adrenal glands. Ten days post-ovariectomy, 30-day-old Sprague–Dawley OVX rats (n = 8-28 rats/group) received oral vehicle (no progestogen), progesterone (10 mg/rat/day), NOMAC (0.3-3.0 mg/rat/day), or MPA (0.1-3.0 mg/rat/day) for 14 days. A fifth non-OVX group received oral vehicle. On Day 15, plasma corticosterone was determined in 5 rats/group and, in another 5 rats/group, adrenal glands were removed and examined for morphological changes. The adrenal glands were removed and weighed in the remaining rats. Comparisons among groups were made using ANOVA methods.

Histopathology of the adrenal glands revealed that ovariectomy (OVX controls) caused adrenal hypertrophy (with some lipid accumulation in the adrenal cortex). MPA and progesterone decreased the weight of adrenal glands in OVX rats by a statistically significant margin relative to OVX control rats (Table 2), a finding similar to that of others [32]. In OVX rats receiving oral MPA, a dose-dependent decrease in adrenal weight was observed, associated with hypotrophy of the zona fasciculata and zona glomerulosa and moderate lipid accumulation in the adrenal cortex; these are changes consistent with inhibition of adrenocorticotropic hormone secretion. In contrast to MPA, NOMAC caused a modest but significant increase in adrenal weight that was associated with adrenal hypertrophy and decreased lipid accumulation in the adrenal cortex. No statistically significant changes in plasma corticosterone were observed between OVX control rats and rats receiving the test compounds. In conclusion, these data indicate that oral NOMAC administration to OVX rats significantly increased adrenal gland weight relative to non-treated OVX rats that was not due to changes in plasma corticosterone.

**Table 2:** Effect of progesterone, NOMAC, and MPA on the pituitary-adrenal axis in OVX rats

**Treatment**	**Dose (mg/rat/day)**	**Rats (n)**	**Adrenal weight (mg)**
Control (No OVX)	–	16	46.59 ± 1.51*
Control (OVX)	–	28	53.32 ± 1.63
Progesterone (OVX)	10	10	39.98 ± 2.15*
NOMAC (OVX)	0.3	10	58.38 ± 3.53
	1	19	60.28 ± 1.89*
	3	9	59.43 ± 2.03*
MPA (OVX)	0.1	8	47.28 ± 2.66*
	0.3	10	37.35 ± 1.77*
	1	8	27.58 ± 1.15*
	3	8	20.86 ± 1.14*

Adrenal weight is shown as the mean ± the standard error of the mean.

*ANOVA*, analysis of variance; *MPA*, medroxyprogesterone acetate; *NOMAC*, nomegestrol acetate; *OVX*, ovariectomized.

* *P*<0.05 vs OVX control (ANOVA).

To directly test for anti-inflammatory activity, the effects of NOMAC, hydrocortisone, and MPA on cotton/wool-stimulated inflammatory responses in rats were investigated. Sprague–Dawley male rats (weight, 80-100 g; n = 8 per group) were placed on one of the following once-daily oral dosing regimens for 10 days: vehicle (negative control), NOMAC (2.5-10 mg/kg), or MPA (2.5-10 mg/kg). On Day 7, a cotton/wool pellet was implanted subcutaneously in all rats to induce an inflammatory response. On Days 7-10, 2 groups of vehicle-treated rats received twice-daily, fixed oral doses of the glucocorticoid hydrocortisone (10 or 80 mg/kg). On Day 10, rats in all groups were sacrificed, and the adrenal glands and thymus were removed and weighed. Granulomas that had formed around the cotton/wool pellets were removed, dried for 24 hours in an oven (80°C), and weighed. In one rat per group, implantation zones around the pellet, thymus, and adrenals were removed, placed in Bouin’s fixative for 48 hours, processed, and examined with histopathological methods for signs of vascular, cellular, and fibrous reactions (indicative of inflammation).

Hydrocortisone (10 and 80 mg/kg) significantly reduced the weight of the adrenal glands by –26% and –36%, respectively (Table 3, *P*<0.001). The weight of the thymus was also decreased in rats that received hydrocortisone (10 and 80 mg/kg) by –41% and –77%, compared with control rats (*P*<0.001). Unlike hydrocortisone, NOMAC did not cause significant weight reductions in adrenal glands or thymus. No anti-inflammatory activity or adrenal cortex atrophy was observed in animals that received the lowest dose of NOMAC (2.5 mg/kg). At all dosage levels, MPA caused a statistically significant (*P*<0.01) reduction in adrenal gland weight. The highest dose of MPA (10 mg/kg) decreased the weight of the thymus significantly (*P*<0.05).

**Table 3:** Effect of hydrocortisone, NOMAC, and MPA on inflammatory response

**Treatment**	**Dose (mg/kg)**	**Adrenal weight (mg)**	**Thymus weight (mg)**	**Granuloma weight (mg)**
Vehicle	–	38.8 ± 1.4	607 ± 34	29.7 ± 1.5
Hydrocortisone	10	28.6 ± 1.1*	355 ± 42*	24.6 ± 1.4*
	80	25.0 ± 0.9*	139 ± 15*	22.0 ± 1.4*
NOMAC	2.5	36.2 ± 2.1	614 ± 43	23.3 ± 1.4*
	5	35.8 ± 1.7	586 ± 28	27.1 ± 2.5
	10	36.7 ± 1.0	576 ± 46	26.4 ± 1.6
MPA	2.5	32.4 ± 1.0*	636 ± 25	25.4 ± 1.7
	5	30.4 ± 1.5*	624 ± 42	25.8 ± 2.1
	10	24.2 ± 1.4*	512 ± 20*	24.6 ± 1.9*

Sample weights (mg) are shown as the mean ± standard error of the mean. *ANOVA*, analysis of variance; *MPA*, medroxyprogesterone acetate; *NOMAC*, nomegestrol acetate; * *P*<0.05 compared with the vehicle control group (ANOVA).

Granulomas indicative of inflammation formed around the cotton/wool pellet in all groups. Hydrocortisone (10 and 80 mg/kg) inhibited the growth of granuloma tissue around the cotton/wool implants by 18% and 27%, respectively. In rats that received NOMAC, dose-dependent inhibition of inflammation was not observed. At the lowest dose (2.5 mg/kg), NOMAC inhibited the development of granuloma tissue significantly (*P*<0.01), but larger doses of NOMAC (≥5.0 mg/kg) had no significant effect on granuloma tissue weight (*P*≥0.05). The highest dose of MPA (10 mg/kg) significantly reduced (*P*<0.05) granuloma tissue weight. Although a low dose of NOMAC (2.5 mg/kg) inhibited granuloma formation, histopathology did not reveal signs indicative of anti-inflammatory activity or adrenal atrophy. In summary, these data suggest that NOMAC does not inhibit the pituitary-adrenal axis and has minimal (if any) direct anti-inflammatory or apparent glucocorticoid activity.

### Central nervous system

Ovariectomy significantly reduces circulating as well as brain concentrations of hormonal factors known to effect mood, such as allopregnanolone and beta-endorphin [33,34]. In female OVX Wistar rats, oral NOMAC (0.5 and 1 mg/kg/day) significantly increased allopregnanolone in the hippocampus, but not in other areas of the brain [34]. NOMAC (1 mg/kg/day) also increased beta-endorphin in the hippocampus and NOMAC (0.5 and 1 mg/kg/day) increased beta-endorphin in the hypothalamus. In the same study, estradiol valerate (0.05 mg/kg/day) alone increased allopregnanolone and beta-endorphin in OVX rats. Combining 1 mg/kg/day NOMAC and estradiol valerate increased allopregnanolone concentrations to values higher than with either drug alone. Coadminstra-tion of NOMAC (1 mg/day) and estradiol valerate increased beta-endorphin values significantly in hippocampus, frontal, parietal, and hypothalamic areas of the brain, as well as plasma to values higher than with either drug alone, and to levels similar to those observed in unoperated controls. Coadministration of lower doses of NOMAC (0.05, 0.1, 0.2, 0.5 mg/kg/day) and estradiol valerate did not augment brain concentrations of beta-endorphin. The effect of NOMAC in this OVX rat model system to influence allopregnanolone and beta-endorphin was different from other progestogens, including progesterone, DRSP, MPA, and dydrogesterone [33-35].

### Mineralocorticoid and antimineralocorticoid activity

Mineralocorticoids (including aldosterone) deserve special attention because they have been shown to interact with progesterone to alter renal function in rats [36]. In the presence of mineralocorticoid hormones, progesterone increases the loss of sodium and chloride in urine and inhibits aldosterone binding to mineralocorticoid receptors (MRs) [36]. Progesterone [36-38] and NOMAC [39] also inhibit ^3^H]-aldosterone binding to rat kidney MRs. In contrast, derivatives of 17alpha-hydroxyprogesterone (such as MPA and MAC) do not inhibit the binding of ^3^H]-aldosterone to rat kidney MRs [11].

In 3 previously unpublished studies, the antimineralocorticoid activity of oral NOMAC was assessed in OVX rats using a method similar to one described previously [40]. In all 3 studies, Wistar rats underwent bilateral ovariectomy to decrease the concentration of endogenous progesterone (antimineralocorticoid). After a 1-week recovery period, urine was collected overnight for baseline assessment (sampling time: t_0_) of urinary sodium [Na^+^ and potassium [K^+^. On Day 8, rats received a placebo (oral vehicle), NOMAC, or reference compound (spironolactone or DRSP) in the morning and urine was collected overnight (sampling time: t_1_). On Days 9 and 10, rats received the same compound they received during the morning of Day 8. Urine was also collected during the night of Day 10 (sampling time: t_3_).

**Figure 5:**
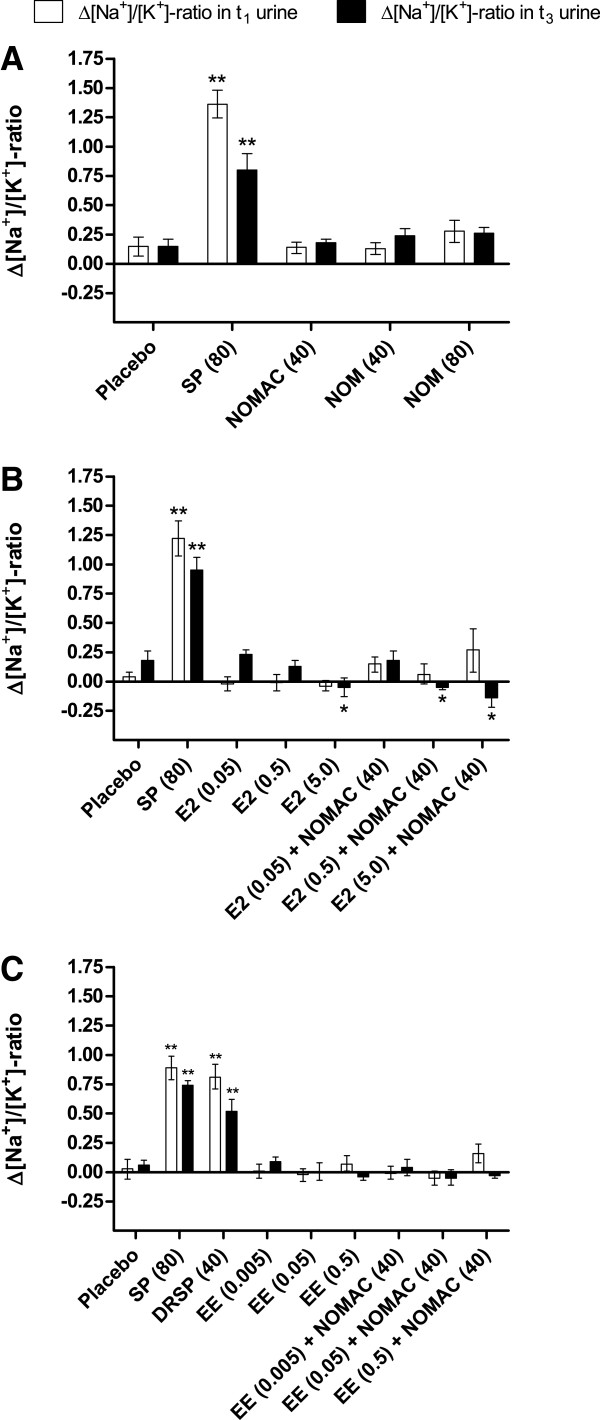
**Mean urinary change in sodium/potassium (Δ[Na^+^]/[K^+^]) ratio in rats after oral administration of (A) spironolactone (SP), nomegestrol acetate (NOMAC), or nomegestrol (NOM); (B) SP, 17β-estradiol (E2), or NOMAC/E2; and (C) SP, drospirenone (DRSP), ethinyl estradiol (EE), or NOMAC/EE.** t_1_, overnight urine; t_3_, urine collected during last time. Doses are in mg/kg. Results are shown as mean ± standard error of the mean. * *P*<0.05; ** *P*<0.01 indicates the results were statistically significant compared with placebo-treated animals (analysis of variance).

In the first experiment, oral NOMAC (40 and 80 mg/kg) and oral nomegestrol (80 mg/kg) were tested alone for antimineralocorticoid activity, and spironolactone (80 mg/kg) was used as a reference antimineralocorticoid compound. Rats receiving spironolactone showed a significant increase in the mean change (Δ)[Na^+^]/[K^+^] ratio at t_1_ and, to a lesser extent, at t_3_, when compared with placebo treatment, confirming that spironolactone possessed antimineralocorticoid activity (Figure 5A). In contrast, the mean Δ[Na^+^]/[K^+^] ratio remained similar to placebo at t_1_ and t_3_ after treatment with NOMAC or nomegestrol, suggesting that NOMAC and nomegestrol did not have antimineralocorticoid activity. In the second experiment, the effect of E2 alone and in combination with NOMAC was evaluated. As expected, spironolactone (80 mg/kg) again caused a significant increase in the mean Δ[Na^+^]/[K^+^] ratio at t_1_ and t_3_ compared with placebo (Figure 5B). In animals receiving E2 alone, a dose-dependent decrease in the mean Δ[Na^+^]/[K^+^] ratio was observed at t_3_ (statistically significant at 5 mg/kg E2). At t_3_, rats that received 40 mg/kg NOMAC and 0.5 or 5 mg/kg E2 also showed a modest but significant decrease in the mean Δ[Na^+^]/[K^+^] ratio, suggesting that NOMAC did not alter the effect of E2. In the third experiment, the effect of the synthetic estrogen, EE, on mineralocorticoid activity was evaluated. In this study, the spironolactone-derived progestogen DRSP (40 mg/kg) and spironolactone (80 mg/kg) were both used as reference compounds and, as expected, both caused similar increases in the mean Δ[Na^+^]/[K^+^] ratio at t_1_ and t_3_ compared with placebo treatment (Figure 5C). In contrast, EE alone (0.005-0.5 mg/kg) or combined with NOMAC (40 mg/kg) did not alter Δ[Na^+^]/[K^+^] ratio at t_1_ or t_3_. Taken together, these 3 studies indicate that NOMAC, administered alone or co-administered with EE or E2, does not have antimineralocorticoid activity.

### Effects on carbohydrate and lipid metabolism

Although progestogens chiefly interact with PR, adipose tissue has been shown to act as an indirect metabolic target of progesterone and E2 [41,42]. Hazard *et al.*[43] compared the effects of equal dosages (5 mg/kg/day) of progesterone, MAC, norethindrone acetate, and NOMAC on lipid metabolism and plasma glucose in adult female rats. MAC, a 17alpha-hydroxyprogesterone derivative, induced significant increases in glucose, total cholesterol, high-density lipoprotein (HDL) cholesterol, triglycerides, and phospholipid plasma levels. After 18 days, a marked increase was observed in the weight of parametrial adipose tissue (PAT) relative to control animals. Animals receiving norethindrone acetate also had significant glycemia at Day 18; lipid parameters were lowered at Day 4, and only triglycerides reverted to normal levels at Day 18. Animals receiving norethindrone acetate had decreased body weight (–9%) relative to control animals, but PAT weight was similar to control animals. In contrast, NOMAC and progesterone did not alter lipid or glucose metabolism or HDL cholesterol, and plasma and tissue lipolysis remained unchanged. Animals receiving NOMAC and progesterone both experienced a similar increase in body weight over time as controls, but only progesterone caused a statistically significant increase in PAT weight (*P*≤0.05) relative to controls at Day 18. In our previously unpublished studies, OVX rats receiving vehicle or oral NOMAC (10 mg/kg/day) for 21 days had similar levels of alkaline phosphatase, plasma glucose, plasma insulin, triglycerides, total cholesterol, and HDL cholesterol (Table 4).

**Table 4:** Effect of 21 days treatment with 10 mg/day NOMAC on mean metabolic parameters in OVX rats compared with intact and OVX controls

	**Alkaline phosphatase (U/L)**	**Glucose (g/L)**	**Insulin (ng/mL)**	**Triglycerides (mmol/L)**	**Total cholesterol (g/L)**	**HDL cholesterol (g/L)**
Intact control	124	1.06	0.61	0.48	0.58*	0.33
OVX control	150	1.04	0.71	0.49	0.70	0.38
NOMAC	156	1.03	0.72	0.54	0.72	0.40

*ANOVA*, analysis of variance; *HDL*, high-density lipoprotein; *NOMAC*, nomegestrol acetate; *OVX*, ovariectomized. *N* = 10 per group; * *P*<0.05 vs OVX control (ANOVA).

Female rats weighing 180 to 200 g were OVX or kept intact 1 week before treatment was started. Rats (*n* = 10) were orally treated with vehicle or 10 mg/kg/day of NOMAC for 3 weeks. Blood samples were collected from fasting animals from the retro-orbital venous sinus under light ether anesthesia 1 hour ±30 minutes after treatment. Plasma samples were stored at –20ºC until measuring the alkaline phosphatase, glucose, insulin, triglycerides, cholesterol, and HDL cholesterol.

In summary, NOMAC administration had little effect on carbohydrate and lipid metabolism in rats. Moreover, NOMAC and E2 administration to nonhuman primates did not alter total cholesterol, HDL, or plasma triglycerides [44,45]. These data have been confirmed in clinical studies [46].

### Effects on bone

Ovariectomy, which can induce osteopenia, can decrease bone mineral density (BMD) in rats [47]. However, little is known about the effect of 19-nor-progesterone (including NOMAC) on BMD and other bone health parameters. To determine the effect of NOMAC on BMD in the rat, sexually mature female rats (21 weeks old; n = 8 per group) underwent OVX and received (1) once-daily subcutaneous injections of E2 (1 μg/kg) for 90 days; (2) once-daily oral NOMAC (1 mg/kg) for 90 days; or (3) once-daily oral NOMAC (1 mg/kg) plus subcutaneous E2 (1 μg/kg) for 90 days. The BMD of the lumbar rachis decreased by 5% in OVX rats, with significant changes observed as early as the first dual-energy X-ray absorptiometry (DEXA) measurement (Table 5). In the intact control group (non-OVX rats), BMD increased over 90 days (+12%). Although E2 was able to restore BMD to a higher level than that observed in OVX control rats, BMD was not fully restored to levels observed in the intact, non-OVX controls. There were no statistically significant differences between the BMDs of OVX rats and rats receiving NOMAC. Importantly, NOMAC did not prevent the restorative effect of E2 on BMD in OVX rats (Table 5).

**Table 5:** Effect of NOMAC on osteodensitometry (DEXA) measurements of L1 to L5 of the lumbar rachis

**Treatment**	**DEXA measurements (%)**			
	**Day 20**	**Day 41**	**Day 62**	**Day 90**
Intact control^†^	104.8 ± 1.4	105.5 ± 2.5	105.9 ± 1.5	108.0 ± 1.4
% treatment of intact control	100	100	100	100
OVX control^†^	94.4 ± 2.3	95.5 ± 1.7	97.0 ± 2.8	95.1 ± 2.7
% treatment of intact control	−9.9	−9.4	−8.4	−12
E2 (1 μg/kg/day)^†^	98.2 ± 0.7	100.8 ± 1.4	103.6 ± 1.7	102.8 ± 1.8
% treatment of OVX control	+4.0	+5.5	+6.8*	+8.1**
NOMAC (1 mg/kg/day)^†^	92.7 ± 0.8	93.4 ± 0.5	92.2 ± 1.1	93.2 ± 2.3
% treatment of OVX control	−1.8	−2.3	−4.9	−2.0
E2 (1 μg/kg/day) + NOMAC (1 mg/kg/day)^†^	98.7 ± 1.1	97.5 ± 1.9	102.0 ± 1.0	101.5 ± 1.1
% treatment of OVX control	+4.6*	+2.1	+5.2*	+6.7*

*ANOVA*, analysis of variance; *DEXA*, dual-energy X-ray absorptiometry; E2, 17β-estradiol; *NOMAC*, nomegestrol acetate; *OVX*, ovariectomized. ^†^ Data are percentages of DEXA values and are expressed as the mean ± standard error of the mean. * *P*<0.05; ** *P*<0.01 compared with OVX control animals (ANOVA).

At 3-week intervals, osteodensitometry measurements of the lumbar vertebrae (L1 to L6) were made using DEXA. After 90 days, the animals were sacrificed, the femur and L5 vertebra removed, and dynamic osteodensitometric measurements were made.

### Effect on endothelial function and the cardiovascular system

The effects of progestogens on endothelial function are complex, partially mediated by progestogen binding to the androgen receptor (AR), ER, GR, or MR [48]. However, the effect of E2 on endothelial function is unequivocal: E2 can enhance the production of endothelial nitric oxide synthase (eNOS), increasing the release of nitric oxide, which can relax vascular smooth muscle, inhibit platelet aggregation, and inhibit the progression of atherosclerosis [49]. In contrast to the effects of E2, the effect of progestogens on endothelium-dependent vasodilation and antiplatelet activity are less well understood. It has been proposed that progestogens with non-androgenic activity and antimineralocorticoid properties are not deleterious to cardiovascular health, especially when used in combination with E2 [48]. NOMAC, which displays moderate anti-androgenic activity, can stimulate nitric oxide synthesis, eNOS activity, and eNOS expression in human endothelial cells derived from human umbilical veins [50] and does not impair estrogen-dependent induction of eNOS [50]. In a comparative study, MPA and progesterone markedly decreased eNOS mRNA and protein in endothelial cells, whereas LNG and NOMAC had minimal effects on eNOS [51].

NOMAC appears to have a neutral effect on cardiovascular function. Intramuscular administration of NOMAC (0.5 or 2.5 mg/kg) over 4 hours in halothane anesthetized male beagle dogs (n = 5 per group) did not produce statistically significant differences in blood pressure, heart rate, femoral blood flow and resistance, carotid flow and resistance, cardiac flow, total peripheral resistance, or maximal ventricular performance when compared with a vehicle-treated control group (data on file with MSD). In female *Cynomolgus* monkeys, supramaximal single oral doses of NOMAC (5, 20, and 80 mg/kg) did not alter hemodynamics (heart rate and arterial blood pressure) or cardiac electrophysiologic parameters (PQ, QRS, QT, and heart rate-corrected QT intervals) over a 6-hour period (data on file with MSD). These doses were much higher than the ID50 reported for ovulation inhibition (0.14 mg/kg) in the same species [13]. Taken together, these studies suggested that NOMAC did not affect parameters of cardiovascular function in dogs and nonhuman primates. NOMAC may have an inhibitory effect on prostaglandin-induced contractions of uterine smooth muscle [52], but any effect on prostanoid-mediated effects in the vasculature is not known.

### Pharmacokinetics and metabolism

NOMAC pharmacokinetics have been investigated most extensively in humans [53,54]. In women, oral administration of 2.5 mg NOMAC per day results in rapid absorption with a time to maximal concentration of approximately 1.5 hours. Elimination half-life is approximately 46 hours and steady state is reached after 5 days of dosing.

Nomegestrol acetate is metabolized by hepatic cytochrome P450 (CYP) system enzymes, including CYP3A3, CYP3A4, and CYP2A6 into metabolites with little or no effect on progesterone receptor activity [55]. As such, there is a possibility for drug interactions when combined with inducers or inhibitors of the CYP system, such as rifampicin or ketoconazole. Excretion is via the urine and feces [55].

## Conclusions

NOMAC, a highly selective contraceptive progestogen derived from 19-nor-progesterone, is a full PR agonist without significant agonistic or antagonistic ER activity. NOMAC binds with strong affinity to PR in hormone-sensitive cells derived from rat, rabbit, or human. It possesses strong antigonadotropic activity (dose-dependent inhibition of ovulation in rats and nonhuman primates) and has indirect, PR-mediated anti-estrogenic effects. In addition, NOMAC does not exhibit androgenic activity and has moderate anti-androgenic activity in rats. It is also devoid of glucocorticoid and antimineralocorticoid activity and does not appear to adversely affect lipid or carbohydrate metabolism, BMD, endothelial function, or hemodynamic parameters in selected animal models. NOMAC combined with E2 has been developed as a new monophasic oral contraceptive [5-10].

## Abbreviations

*B*_*max*_: Maximum concentration of binding sites; BMD: Bone mineral density; CHO: Chinese hamster ovary; CMA: Chlormadinone acetate; COC: Combined oral contraceptives; DEXA: Dual-energy X-ray absorptiometry; DNG: Dienogest; DRSP: Drospirenone; E1S: Estrone sulfate; E2: 17β-estradiol; EE: Ethinylestradiol; eNOS: Endothelial nitric oxide synthase; hAR: Human androgen receptor; HDL: High-density lipoprotein cholesterol; hER_alpha_: Human estrogen receptor α; hER_beta_: Human estrogen receptor β; hGR: Human glucocorticoid receptor; hMR: Human mineralocorticoid receptor; hPRB: Human progesterone receptor B; IC50: Half maximal inhibitory concentration; ID50: 50% inhibition dose; *k*_*d*_: Equilibrium; *k*_*−1*_: Dissociation rate constant; LNG: Levonorgestrel; MAC: Megestrol acetate; MPA: Medroxyprogesterone acetate; MR: Mineralocorticoid receptor; [Na^+^]/[K^+^]: Sodium/potassium ratio; NETAC: Norethisterone acetate; NOMAC: Nomegestrol acetate; OVX: Ovariectomized; PAT: Parametrial adipose tissue; PR: Progesterone receptor; RBA: Relative binding affinity; SD: Standard deviation.

## Competing interests

The author was an employee of MSD, Oss, The Netherlands, when this work was performed. He has no other competing interests.

## Author’s contributions

HvD participated as an investigator in a number of the described studies done by Organon, Schering-Plough, and most recently, Merck. He made substantial contributions to analysis and interpretation of data, drafting the manuscript, revising critically for intellectual content, and approved the final manuscript.

## Financial disclosure

Merck Sharp & Dohme Corp., a subsidiary of Merck & Co., Inc., Whitehouse Station, NJ, USA, provided financial support for the studies described.
